# The Effect of Employee Competency and Organizational Culture on Employees’ Perceived Stress for Better Workplace

**DOI:** 10.3390/ijerph19084428

**Published:** 2022-04-07

**Authors:** Jina Kim, Hye-Sun Jung

**Affiliations:** 1 Department of Public Health, Graduate School, The Catholic University of Korea, Seoul 06591, Korea; ggomjina@naver.com; 2 Department of Preventive Medicine, College of Medicine, The Catholic University of Korea, Seoul 06591, Korea

**Keywords:** organizational culture, employee competency, employee perceived stress

## Abstract

Although the meaningful relationship between organizational culture and employee performance is a widely-researched topic, there is scant research available how organizational culture impacts on employees’ perceived stress in the workplace, affecting their performance. This might cause a difficulty to guide practitioners as to what organizational elements can be applied to reduce employee perceived stress. To add the level of robustness and fill the gap in the literature, the present research explores the effect of organizational culture with employee competency on workers’ perceived stress which has emerged as a common occupational disease and affected employees psychologically and physically; thus, affecting their performance. Using 641 responses, the statistical findings of the present research insists that HR practitioners should match the type of organizational culture and employee competency situationally to reduce employee stress. The current authors suggest that organizations desiring the adaptability competencies (Professional competency) for their employees should build a clan organizational culture. In contrast, organizations should encourage a market organizational culture for their employees who possess customer orientation competency (Simply result-oriented competency). The research outcomes provide additional knowledge to the existing literature, enhance academicians’ understanding of the research topic, and serve as a significant knowledge base for further empirical research.

## 1. Introduction

Organizational culture is the way employees perform assigned tasks and interact with others in the organization. Besides, it refers to symbols and values understood and adhered to by everyone in the organization [1,2] Organizational culture as can also be understood as individuals’ mindset that makes them distinct from others [3]. Thus, it is crucial in determining how employees perform in a company. Unlike other factors like lack of incentives, organizational culture might significantly impact workers’ stress [4].

Organizational culture is vital to employee performance. Performance is what employees do or do not do [5]. Employee performance is one of the most significant elements in an organization since it increases the organization’s efficiency and effectiveness [6]. Organizational culture represents the collective values, principles, and beliefs of corporate members [7]. Precisely, the culture with the company determines how employees perform and their engagement at the workplace. An organizations’ culture affects employee performance since it proposes to employees how to operate [8]. A strong organizational culture allows for open communication and participation in the decision-making. Accordingly, Shahzad [8] noted that employee participation, innovation and risk-taking, reward system, the openness of communication, and customer service orientation are essential parameters in understanding organizational culture’s impact on employee performance. Employee participation increases goal acceptance, and it entails delegating tasks based on individual’s responsibilities; thus, influencing their performance [9].

Prior literature has also mentioned that not only organizational cultures, but worker’s perceived job stress also contributes to organizational inefficiency, impacting on high staff turnover, absenteeism and finally, decreasing the quality and quantity of job performance thus, causing low job satisfaction [10,11]. This implies that employee job stress may cause worker’s burnout with serious reduced performance and employees are needed immediate social support [12]. Jamal [13] has proven that there is little doubt that job stress factors can clearly reduce the organizational profit due to low level of job performance. Prior studies have supported the negative linear relationship between the measures of job stress and performance [14,15].

In the perspective of employee competency, employee adaptability is one of the core competencies of employees which might affect their performance. Precisely, it involves an adaptive response to deal with new environmental situations. Adaptability is the employee’s ability to adapt to changes [16]. Besides, adaptability is how individuals cope with change and respond to dynamic environments [17]. Employees with adaptability competency tend to be flexible in dealing with diverse situations and thus, this competency is an increasingly important performance dimension in dynamic contexts than other individual competencies [1,2]. Also, they do not hesitate to cooperate with others to complete tasks accordingly. Workers who have improved adaptability capabilities could overcome their difficult and complex jobs and feel satisfied with their present situations [18]. Clan culture emphasizes flexibility, implying that it is in line with employee adaptability competency. Precisely, clan culture enhances workforce flexibility. Kang and Lee [1] have insisted that adaptability capabilities might be connected with attributes of clan culture. Thus, clan culture supports employee adaptability competency. Contrarily, employee adaptability competency negatively relates to market culture. A market culture stresses stability and individuality, where every person pursues their interests. This culture does not support adaptability since it does not encourage employees to be flexible and cooperate with others to attain the best outcomes.

Customer orientation competency entails serving and helping customers to meet specific demands. Besides, it involves reading the customer facet, delivering service, and keeping customers informed [19]. With this competency, employees can ascertain customer needs and find the most appropriate way to satisfy such needs. The current study selected this competency to use one of main factors for this research based on prior studies [1,2] which suggested a positive relationship between employee customer orientation competency and market culture. Accordingly, product capability and customer orientation significantly correlate, suggesting a positive relationship between market culture and customer orientation [2]. The market culture encourages individuality among employees, implying that they can work independently and satisfy customer needs as required. However, clan culture emphasizes cooperation and flexibility among employees; thus, negatively relating to customer orientation competency.

Previous studies have widely explored the organizational culture and employee competency. For instance, The prior studies [1,2] have extensively investigated different employee competencies and corporate culture, specifically focusing on market and clan organizational cultures. The current research explores the effect of corporate culture and employee competency on workers’ stress. Worker’s stress has emerged as a common occupational disease, which has affected employees psychologically and physically; thus, affecting their performance [20]. Besides, job stress affects employee outcomes and satisfaction [21]. Job stress occurs due to many factors, such as workload, lack of incentives, and motivation [22]. Besides, there is a need to explore the importance of employees’ stress management to enhance workers’ performance. Hence, this research provides additional insight into the existing knowledge on the employees’ job stress between organizational culture and workers’ competency, suspecting reasonably employees who have an adaptability competency could be more stressful within market culture than within clan culture and in contrast, customer-oriented workers could be less stressful within market corporate culture than within clan culture.

## 2. Literature Review

### 2.1. The Importance of Organizational Culture to Improve Employee’s Job Performance

Organizational culture delineates task completion and employee interaction within an organization. Organizational culture shapes how firms operate and perform [23]. Abu Khadar [24] associates culture with the various values, beliefs, symbols, and rituals that direct the functioning style of people with an entity. Apart from binding employees, organizational culture offers direct companies. Notably, the most challenging task for any company during change may be to transform its culture. Employees may be accustomed to a particular way of working, making it difficult to adjust to new requirements. According to Thi and his colleagues [25], organizational culture relies on various factors such as company objectives, management style, employee belief system, and operation environment. Therefore, many corporate cultures ranging from well-structured and highly bureaucratic companies to collaborative ones exist [25]. Although these cultures have varying effects on motivation levels and performance, employees tend to work harder to accomplish company goals when they consider themselves part of the organizational environment.

This paper focuses on the clan and market cultures to define how organizational culture and employee competency affect worker stress. The clan culture delineates organizations that operate like large families or tribes. Here, members have similar values and chase the same objectives. This culture highly values connection and consensus within the group and tends to de-emphasize competition. Fakhri and his associates [26] argue that the clan culture allows all team members to feel supported and valued. These feelings enable employees to pursue individual initiatives without feeling isolated. Moreover, they enable empowerment to thrive, although issues related to compromising an organization’s creative potential have been raised with this culture’s use. Conversely, market culture underscores accomplishment at an individual level and encourages competitiveness [27]. In this culture, personal performance is usually the most substantial factor in determining advancement, termination, and compensation decisions.

Employee job performance usually depends on the ability of organizations to implement the proper cultural foundation and clear priorities. Addullahi et al.’s [28] study on the Malaysian educational sector reveals that organizational culture drives job performance by encouraging innovation. Healthy and well-realized cultures tend to attract better and more often innovation than their counterparts with environments that do not value employees [28]. Healthy organizational cultures result in an established pool of professionals who can tackle problems in creative ways. Saha and Kumar [29] support these findings by describing corporate culture as the moderator of job satisfaction and affective commitment among employees. Highly-satisfied employees tend to be more committed to the success of their organizations and their colleagues.

Organizational culture also improves employee job performance by emphasizing shared goals and values. According to the previous study [30], a strong company culture implies observing corporate values and mission. A robust culture generates employees with a well-tuned direction sense and facilitates the creation of standard success definitions to allow organizations to develop as teams. Similarly, Narayana [31] argues that successful companies have cultures centered on decisively held and widely-shared beliefs reinforced by structure and strategy. Employees in such environments recognize how executive management requires them to address any situation and consider the expected reaction to being the appropriate ones [31]. They also understand the concept of reward for employees who demonstrate organizational values. Happy employees are more productive and highly engaged in the workplace [30]. Organizations with more engaged workers tend to have more revenues compared to those with less-involved ones.

### 2.2. Prior Studies, Which Have Already Provided the Meaningful Relationships between Organizational Culture and Employee Performance

The meaningful relationship between organizational culture and employee performance is a widely-researched topic. Studies such as Christine [32] help demonstrate this relationship by defining employees as a critical factor in guaranteeing longstanding organizational success and survival. By focusing on Hilton Hotel, United Kingdom, this study indicates that organizations with robust cultures benefit from positive environments that foster unity, uniformity, identity, engagement, and enthusiasm [32]. These aspects play a critical part in enhancing job satisfaction and worker’s capability. Organizational culture mainly involves cognitive systems explaining how workers reason and make judgments [33]. These systems also govern communication among employees and with external stakeholders. Although these systems are intangible, companies seeking to improve employee productivity and performance must address them as the first concern [34,35]. Positive cultures, especially those involving rewards, compensation, training, administrative support, growth opportunities, and communication, encourage increased employee performance.

Kang and Lee [1] support these findings by emphasizing the need for organizations to develop an employee compensation strategy to achieve a sustainable competitive advantage. Since the role of employees in establishing sustainable competitive advantage is unquestionable, organizations today are increasingly relying on compensation strategies to motivate employee performance. While some researchers have criticized the effectiveness of compensation for encouraging employee performance due to its short-term nature, others support this strategy as a critical aspect of positive organizational culture. Richard and Kang [2] also note this disagreement among academics about whether organizations should use compensation programs as a strategy to encourage employee performance. These findings help demonstrate why organizations should incorporate various strategies as part of their organizational culture instead of relying on compensation tactics alone since they can be counterproductive in some instances.

Modern-day organizations endeavor to realize profitability, fast growth, continued improvement, and future preparation. Despite this desire, working in a constantly-changing environment makes it challenging for companies to predict these changes [36]. This unpredictability has resulted in a situation whereby businesses dedicate extensive resources to achieve high performance. Organizations need to identify the factors impacting performance to achieve high productivity. In their study on the linkage between corporate culture and job enjoyment, Maswani and Rina [4] established that a strong culture is a key to good performance. Positive and robust corporate cultures can encourage brilliant individual performance. Conversely, weak and negative cultures may cause demotivation and dissuade outstanding employees from meeting their potential [7,35]. These findings demonstrate organizational culture’s direct and active part in performance management.

The prior study [37] defined consistency, involvement, mission, and adaptability as the four critical traits of corporate cultures. Organizations with an influential culture empower their people and build their working environment around teamwork and expanding human capacity [38]. Consistency, the ability to adapt to shifting business environments, and create change, separate companies with a solid organizational culture from weak ones [37]. Employees work hard in environments that have a clear sense of direction and purpose. Well-coordinated, integrated, and highly consistent environments are a powerful stability source for employees [38]. Employee performance in such environments often occurs in the form of greater productivity, higher customer satisfaction levels, reduced turnover, lower absenteeism levels, and higher customer satisfaction rates.

### 2.3. The Positive Relationship between Adaptability Competency and Clan Culture

The primary focus of the clan organizational culture is mentorship and teamwork. According to Kerr and Slocum [3], organizations guided by this culture value flexibility, discretion, integration, and internal focus. This culture is not only people-focused but also treats a company like a single big and happy family. The highly collaborative nature of this culture implies that each person is evaluated, and the relationship among employees is of top precedence. Clan culture-based companies tend to be action-oriented, highly flexible, and adaptable to change [39]. In such cultures, organizations survive and thrive in collaboration, loyalty, and tradition. At times denoted a collaborative culture, clan-based organizations tend to have great concern and affiliation with teamwork, collaboration, and participation.

Due to its highly flexible nature, the clan organizational culture tends to have a positive relationship with employee adaptability competency. Park & Park [40] define employee adaptability as the capacity to adjust to changes within the work environment. The level of adaptability competence can support positive outcomes related to increased work capability and career success. This competency can also facilitate organizational results, such as learning, change management, and sustaining shifting customer expectations [40]. According to Gorzelany et al. [41], clan cultures have high employee engagement levels. The culture’s highly adaptable nature implies that employees have an increased possibility for achieving market growth and pursuing personal initiatives like further education. Clan cultures tend to work well in companies where a large employee percentage work remotely [39]. In such environments, employee adaptability competency thrives since companies create a communicative and empathetic culture.

### 2.4. The Negative Relationship between Adaptability Competency and Market Culture

Unlike the clan culture, which underscores collaboration and mentorship, competition and growth are the primary focus in market-cultured organizations. According to Ali et al. [42], the market organizational culture focuses on stability, control, and external fixation. Companies with this culture prioritize profitability and evaluate every activity in the workplace with the bottom line in mind. Likewise, each position in such organizations possesses a goal that supports the overall or more significant objective. Na et al. [43] noted that there are usually several separation degrees between governance roles and employees in this culture. Due to the results-oriented nature of market-cultured organizations, they tend to emphasize external success instead of internal satisfaction [42]. A market-oriented culture emphasizes the significance of achieving results, meeting quotas, and reaching targets [44]. Such cultures also tend to rely on compensation-based strategies to encourage employee performance primarily.

Market organizational cultures are usually profitable and successful due to their external focus. Despite their extensive success, organizations with this culture tend to affect employee adaptability competency negatively. Due to the pressure of working hard to meet external objectives, this culture may make it challenging for employees to engage with their work meaningfully [45]. Pressure may, in turn, translate to workplace stress and have adverse repercussions on the mental welfare of workers. Workplace stress increases the risk of burnout, depression, anxiety, and substance abuse disorders among employees [45]. All these issues can drive employees out of the workplace due to increased absenteeism rates and high turnover. Adaptability competency implies having flexibility in addressing change, the capacity to handle multiple demands, embrace new situations with innovative or fresh ideas [44]. The pressure associated with market organizational cultures may compromise adaptability competency and the capacity of employees to achieve a professional purpose.

### 2.5. The Positive Relationship between Customer Orientation Competency and Market Culture

Companies with an advanced market culture promote close affiliations with customers, suppliers, unions, and contractors, improving employee customer orientation [1,46]. According to Racela [47], customer orientation in both service and product organizations depends on organizational capabilities, such as customer relating, market sensing, and customer response. According to the previous study [48], market culture is related to organizations that focuses on gaining competitive advantages. Therefore, a market culture within an organization enables the company to detect the changing market trends, retain and develop close relationships with the consumers, and satisfy the client’s needs through customer-response expertise.

Employee customer orientation, another form of employee competency, largely depends on the effectiveness of market corporate culture. Precisely, the product capacity that a company provides to its customers and the ability of these products to meet or exceed the client’s expectations serve as the significant proxy for customer orientation. According to Richard and Kang [2], companies with a market corporate culture produce and sell goods that address the customers’ needs and expectations. The existing corporate culture serves as an originator of market coordination. Companies with advanced levels of sooq orientation create an organizational culture that promotes trust, supports corporate-wide associations, and leverages the members’ capabilities and experiences [49]. Previous studies [50,51] describe market culture as the corporate-wide generation of market aptitude regarding the present and future consumer needs, organization-wide responsiveness, and dissemination of intelligence across the business. Hence, a market culture within an organization helps organizations deliver more excellent customer value by meeting the client’s needs and demands.

### 2.6. The Negative Relationship between Customer Orientation Competency and Clan Culture

Clan corporate culture adversely affects the employees’ focus in understanding the customer’s changing needs and wants. According to Kang and Lee [1], clan corporate culture contain some sense of tradition and group loyalty, focuses more on flexibility, discretion, and focuses primarily on the growth and acquisition of new resources. Besides, Tasgit et al. [52] denote a negative correlation between clan culture and customer orientation by describing this type of organizational culture as focusing on internal values and issues other than the needs and development of the external environment. A scientific study by Gao [53] found that clan cultures contain unique attributes, such as interpersonal cohesiveness, loyalty, and tradition, which results in a lack of attention in managing market needs and adversely influences market orientation.

Besides, Xiong and his associates [54] argue that organizations with clan corporative culture stress cohesiveness and teamwork, which improves the employee’s social performance and corporate achievements but does not encourage the employees to understand and meet the changing consumer needs. A work environment that stresses more on familial or class associations gives less focus to the consumer since the employees establish close relationships among themselves, a concept that raises a poor attitude in fulfilling customer needs [55,56]. Thus, organizations that primarily rely on clan corporate culture find it hard to meet the needs of the changing market.

### 2.7. Employee’s Stress Management and Its Relationship with Culture and Competency

Stress is a physical and mental condition that affects a person’s effectiveness, productivity, health, and quality of work. Indeed, perceived worker’s stress makes workers to decrease their job satisfaction seriously and reduced quality of worker’s performance. Workplace stress emerges from different factors, such as workplace conflict, family issues, role ambiguity, work overload, and a hostile working environment [20,57]. Whatever the cause of stress is within the workplace, high-stress levels influence the employees’ engagement levels, burnout, and performance. Hence, organizations need to have well-defined interventions to reduce the impact of workplace stressors on the employee’s well-being and productivity.

Effective stress management in any organization is a sure way to increase the employees’ productivity at any given time. Workplace stress management strategies like seminars on job burnouts, training, yoga, supportive corporate culture, affiliations between co-workers, and celebrations affect the employees’ efficiency levels in different positions. According to Patro and Kumar [58], engagement in all these stress management strategies positively influences the employee’s productivity levels, reduces labor turnover, improves interpersonal relations, reduces absenteeism, and promotes physical and mental health. In another quantitative meta-analysis based on 43 primary studies, the prior researchers [59] found that the use of flextime, telecommuting, cognitive-behavioral skills education, and other relaxation techniques to manage stress led to greater job satisfaction, improved psychological health, reduced absenteeism, and more excellent job satisfaction rates [60,61]. Effective stress management in any organization causes enhanced employee performance through improved employee effectiveness and efficiency [62]. Employees with more incredible stress management skills show more extraordinary skills in accomplishing corporate goals with minimum resources.

The present authors insist that to reduce the perceived work-related stress for workers, practitioners should consider an alignment of factors inside the firm to insure a work stress complimentary fit to those factors. This study contends that perceived stress management should be aligned with the organizational cultures and employee competencies. According to previous studies [1,2,63], employee adaptability competency is more matched with clan cultural attributes than market cultural attributes because the use of clan culture within an organization encourages knowledge creation and flexibility through employee development, promoting employee’s adaptability unlike market culture, which is suitable for simple and inflexible jobs within quite competitive circumstance. In addition, found to be conducive to creativity is the organizational encouragement inherent in a clan culture. Thus, organizations with influential clan culture promote employee adaptability competency and might reduce the prevalence of work-related stress among adaptable employees more than within market culture. Based on the findings of the past studies, the current researchers reasonably anticipate that clan culture is more reliable strategy to augment the employee’s adaptability competency and reduce their chances of experiencing workplace stress than market culture.

**Hypothesis** **1:** *Employees with adaptability competence within a clan culture have lower levels of workplace stress than within a market corporate culture*.

The present study also anticipates that employees in organizations with more incredible market culture tend to be less stressed than clan culture due to the greater mastery of customer orientation competency. Based on the findings of the past studies [1,2,63], market culture supports employee customer orientation competency, making the workers more competent to deal with workplace stressors. Besides, other scholars like Lagrosen and Lagrosen [64] argue that although employee customer orientation competency can increase the demand for corporate services and products, the practice also mitigates the stress that these demands create. Alternatively, customer orientation competency increases the demand without enhancing workplace stress due to increased control. Hence, the current researchers postulates that market corporate culture promotes greater customer orientation competency among employees, consequently reducing more work-related stress due to greater control of the business processes than within clan culture environment.

**Hypothesis** **2:** *Greater customer orientation competency among employees within a market culture makes the employees less stressed than within clan culture*.

## 3. Methodology (Research Design)

### 3.1. Survey Items (Variables)

When it comes to earlier research on organizational factors to improve workers’ performance, reducing their perceived stress, there is little assistance to HR practitioners how they can apply their corporate cultures and competencies that employees possess for boosting employee performance with lower levels of stress. The current authors try to add an insight into HR literature, collaborating existing studies and adding new stress factor. For achieving this goal, the total survey items were used 32 questions to gather participants’ responses. In more detail, regarding two organizational cultures, the survey instrument contained total 12 questions (Clan: 6 items and market 6 items) based on previous studies [1,3]. To measure the two employee competencies, the instrument also included total 10 questions (Adaptability competency: 6 questions and Customer-orientation questions: 4 questions) based on existing studies [1,63]. Finally, perceived stress scale was investigated by 10 questions based on the prior study [65]. All items were borrowed by previous studies which already showed a high degree of reliability and validity (See the Table 1 and Table 2).

### 3.2. Data Collection Procedure and Analysis

In getting the aim of the contemporary research, the present authors conducted two kinds of statistical tools which are (1) SPSS 27 and AMOS 24.0 to measure that how particular employee competencies and corporate cultures affect workplace stress of employees. The authors could gauge the internal consistency of key factors and aptitude influences. Moreover, the author carried out the confirmatory factor analysis (CFA) to measure the validity concerning three major concepts through the suitability of organizational reckoning modeling and also investigate the discriminant validity to identify the overlapping level for the three key concepts, gauging whether the square root of the AVEs has considerable additional information than correlation coefficients with other concepts, for the closing examination was tested to determine the scaling of the study suggestion by use of an operational equation modeling [66].

Regarding obtaining the real dataset, the present research tried to collect more than five hundred samples between 6 September 2021, to 23 September 2021, with the survey credentials being sent online or through individuals. The questionaries distributed total eight hundreds while only six hundred and sixty-seven responses were given back out of the number. Some obtained survey responses which are determined as bad datasets was discarded because more than 20% of the items in the survey were not answered properly by the survey respondents or some respondents did not enter their answers illogically, marking same numbers for whole questions [1,66]. The detailed procedure of the statistical collection for the current study is shown in the Table 3 and the overall features (Demographic Information) of the participants in this study who gave out feedback are depicted in the Table 4.

To obtain the sample evenly, the present authors hired a professional research agency in South Korea so that they handled the data in a professional manner and provided accurate and high-quality data. The agency collected using a ‘Stratified Random Sampling’ method which classifies participants into similar characteristics groups. This sampling method allows that similar characteristic participants may be put into a same group, making sure that different groups were assigned equally. As a result, the current authors could collect the data which have similar age and gender distribution as requested to the agency.

## 4. Statistical Findings

### 4.1. Descriptive Statistics

When it comes to earlier research on organizational factors to reduce worker’s stress as the procedure of the first statistical analysis, the current author conducted the descriptive statistical analysis which is included by numerous basic measuring statistics such as mean, median, standard deviation. Every main variable was gauged through a seven-point Likert scale measurement (1 = Strongly Disagree − 7 = Strongly Agree) (See the below Table 5 in more details). As seen the Table 5, the mean of two organizational cultures indicated the highest value more than two employee competencies and employee’s workplace stress. Furthermore, Table 6 represents the information divided into four groups by the mean of two competencies to check how the mean of two cultures are different depending on the mean of two competencies.

### 4.2. Reliability Analysis

The data examination results concerning the collection of the primary dataset (*n* = 461) are presented in this part. The information was analyzed using SPSS software version 27 and AMOS 24.0 to determine the association between culture, competency, and perceived stress for employees. All arithmetical conclusions were represented in tables with their interpretations provided in the text. In deciding the interior steadiness that shows a measure of gauge dependability, Cronbach’s alpha value was applied using generally accepted law. For instance, if the Cronbach value is 0.6 or more significant, a set of items is composed as a collection [67]. Generally, the reliability coefficient value was 0.676 for clan culture and 0.707 for market culture. Statistically, reliability of constructs in the exploratory examination can be satisfactory if the trustworthiness is greater than 0.6 and desirable if trustworthiness is great than 0.7. As shown Table 7, not only clan and market culture, the Cronbach’s alpha of two competencies and perceived stress was also higher than 0.6, which means all main factors have a high degree of internal consistency.

### 4.3. Confirmatory Factor Analysis

The current study also tested a confirmatory factor analysis (CFA) to measure a conversion validity to identify whether our three key constructs sensibly clarified the inactive characteristics. Altering acceptability attempts to examine the approximation of things dependably measure the essential idea and can be identified as the character load between the idle and perceived variables. Usually, if the factors are not less than 0.5, one can conclude that there is legitimacy [1,66]. As seen by Table 8, all AVE values indicated greater than 0.5, confirming all our key constructs may be observed correct legality because every construct uncovered more than the relating reference esteem (0.5) [68].

### 4.4. Discriminant Validity

**Figure 1:**
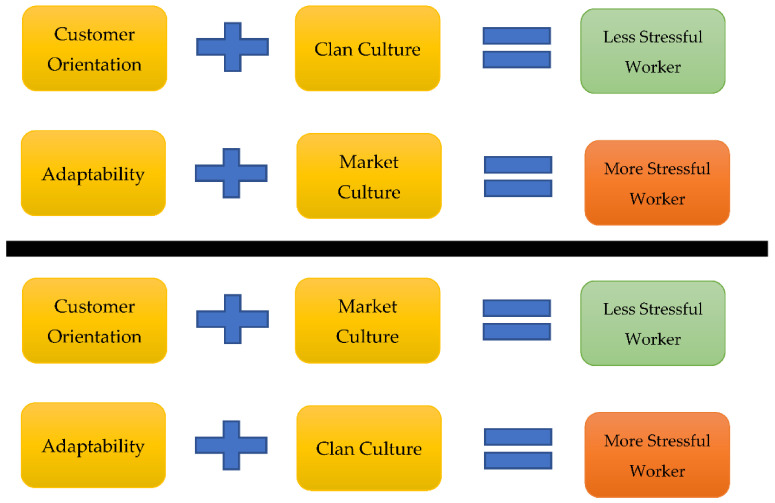
Research Subject of the Present Study.

Our research instrument was gauged by another complex tool to check the quality of construct. That implies that the present research also focused on discriminant validity which points out that if prior theories have not suggested the associations between constructs, they cannot be connected highly with each other through results less or negative association among variables [66,69]. This study generally anticipates less or pessimistic connections between the employee adaptability competency and market cultural characteristics influenced by the administrations based on the past examinations that suggest contradictory directions between corporate clan attributes and corporate market attributes, as already presented in Figure 1. Thus, reasonably, workers who possess an adaptability capability indicates positive linkage with corporate clan attributes and negatively correlated with market culture.

After the convergent validity was measured, the discriminant validity was tried utilizing the Fornell and Lacker [70]. Identifying these negative associations among our main constructs, we could recognize that there exists a strong discriminant validity [1,66,69]. After the convergent validity was measured, the discriminant validity was tried utilizing the Fornell and Lacker [70] law. Theoretically, the discriminant validity investigated the intercorrelation between main factors and noticeable covering figures. As shown in Table 9, the study, based on the Fornell and Lacker’s approach, every value of square roots of AVEs is more significant in every circumstance than the off-slanting components in their comparison line and section. Thus, they indicate that the critical discriminant acceptability has been refined.

### 4.5. Verification Findings for the Path Analysis

**Figure 2:**
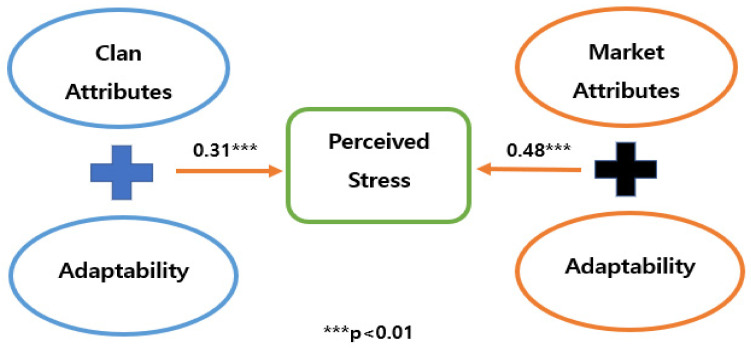
The result of Path Analysis (1).

**Figure 3:**
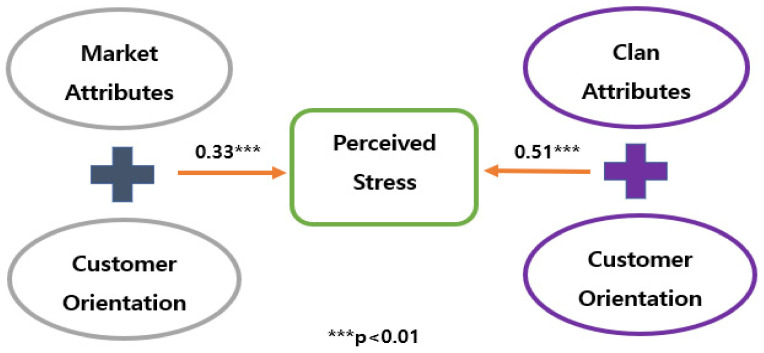
The result of Path Analysis (2).

To identify the research model’s suitableness, this study used RMR, X2, RMSEA, GFI, indicating research model’s fitness. That suggests that the absolute fitness was measured by RMSEA, X2, GFI, RMR, and in terms of the incremental fitness, both TLI and CFI were checked by the current structural equation modeling [71]. Finally, our path analysis showed that employees who have a high degree of adaptability within clan culture attributes were less stressful than within market culture attributes. In contrast, the statistical results also showed that employees who possess a high degree of customer-orientation competency within clan organizational culture are more stressful than within clan organizational culture. According to the statistical findings, fortunately, the first and second hypotheses figured out in the expected directions and therefore, the present authors could accept them (See the Table 10, Figure 2 and Figure 3).

## 5. Implications

### 5.1. Academic Implication

The current research provides the basis for further empirical research on how organizational culture and employee competency impact workers’ stress. The study findings will serve as a significant foundation for the researchers or academicians to base their research. The study will provide valuable insight into the effects of organizational culture and employee competency on workers’ stress. The current research provides detailed information about the link between adaptability and customer orientation employee competencies and clan or market organizational cultures. Besides, the study links these variables to employee performance and worker stress. Therefore, the information will benefit future researchers and academicians who intend to explore similar or related topics. Precisely, they will base their research on the outcomes of the current study, thus reinforcing this study’s findings. In other words, the current research might offer meaningful information relating specific organizational characteristics (culture and competency) with the use of employee stress factors for researchers, who are trying to build their research frameworks that enhance employee performance within less stressful circumstance, thus, contributing to the existing body of knowledge on organizational culture, employee competency impact on workers’ stress. Precisely, it will improve the knowledge and understanding of the different types of corporate cultures and employee competencies and how they affect employee performance and stress.

Also, the study outcomes will enlighten potential scholars about what has already been done on the research topic and the research gap. Hence, the current research will guide scholars on what knowledge gap needs to be filled. For instance, workplace stress is a research area related to the present study, requiring further investigation. Workplace stress is individuals’ response when faced with work demands and pressures that do not match their knowledge and abilities [72]. Workplace stressors refer to conditions that subject employees to stress. These conditions include organizational change, autonomy, difficult relationships at work, job security, workload, and career development [73]. Burnout is one of the factors determining workplace stress and refers to a state of being exhausted psychologically, emotionally, spiritually, and physically [74]. It happens due to chronic stress, characterized by physical and emotional fatigue [75]. Stressful workplaces lead to increased absenteeism, employee turnover, and low productivity [76]. Accordingly, psychological symptoms like worries caused by stress can result in less work productivity [77]. Work stress is related to physical and mental health risks. Workplace stress affects mental health due to job insecurity, low reward, and social support [78]. The high-stress level at the workplace leads to psychological and physical problems among employees [79]. Notably, the negative relationship between job stress and satisfaction determines employees’ perception of meaningful work and engagement in decision-making [21]. Generally, the current research has various academic implications. The study outcomes will provide additional knowledge to the existing literature, enhance academicians’ understanding of the research topic, and serve as a significant knowledge base for further empirical research.

### 5.2. Practical Implication

The present study has several practical implications. First, the outcomes of the present study are significant since it will help the organizations understand employees’ level of job stress by using organizational elements (Culture and Competency) to gain new insights. Workplace stress is one of the major problems affecting the employees’ satisfaction level and performance, subsequently, organizational performance and productivity [20,57]. The adverse impacts of workplace stress need firms to properly manage it and create a conducive environment for all employees. Stress management is crucial in improving employee and organizational performance, which increases productivity and allows for company growth [58]. With the current research outcomes, HR Practitioners can establish effective policies and strategies to manage stress; thus, ensuring that their workplace stress is eliminated.

The present study covers how two employee competencies and work environments influence the level of stress. In this regard, company managers will gain valuable insight into the key stressors; thus, encouraging specific employee competencies, especially those that enable workers to cope with the changes within the organization [1]. Besides, the study findings might benefit practitioners as applying the current study’s findings to design and implement stress management strategies by influencing employee competency and changing the nature of the corporate culture. The current study explored the link between clan and market organizational cultures and workers’ stress. Therefore, the outcomes of this study might allow organizations to create a culture that makes employees feel less stressed and benefit company managers, especially in developing effective policies and strategies to manage stress, which will lead to improved performance and increased productivity.

Additionally, the research findings will allow organizations to address factors affecting employees to improve their performance. Notably, employees’ performances are affected by many issues, including stress, workload, and an unconducive work environment [20,57,62]. A high level of workplace stress is one of the significant factors contributing to poor performance among employees. Likewise, the workload is a significant stressor leading to an unmotivated and disengaged workforce, adversely impacting employee performance. Also, organizations with an unconducive work environment and ineffective cultures tend to experience poor employee performance [2]. The current study explored these factors; hence, the outcomes will be crucial for organizations and company managers to address factors that negatively influence employee performances. As a result, they will have a motivated workforce and subsequently improved performance and productivity.

Further, the current study outcomes will enable companies to prioritize job satisfaction to motivate employees to enhance their performance. Job satisfaction is one of the most significant determinants of employee happiness with their jobs. The study outcomes will enable firms to focus on satisfying their employees; thus, reducing turnover and stress and improving employee and organizational performances. Besides, organizations might benefit from the current study’s findings since they understand how different employee competencies affect workers in terms of stress. With such information, the companies can decide how to train their employees to possess the necessary competencies. Finally, the present study’s findings will allow companies to develop new policies and procedures to reduce the prevalence of occupational stress and improve employee productivity and corporate performance. The study will contribute to the existing knowledge [1,2,3,63,80] by elaborating on employee competency and organizational culture on workers’ focus and productivity. Therefore, the current study’s findings will benefit many business stakeholders from entrepreneurs, managers, employees, private and public businesses.

## 6. Limitation and Recommendation

### 6.1. Limitation

The current study has limitations. The researcher analyzed the relationship between employee performance and organizational culture. Precisely, the past studies focused on the relationships between clan and market corporate cultures with employee adaptability and customer orientation competencies [1,2,63]. Based on this, the present study focused on only two employee competencies to understand how they impact employee stress within organizations with a clan or market culture. The researcher ignored other significant employee competencies that can give in-depth insight into the link between organizational culture and employee performance. Besides, the current research only explored the relationship between corporate culture and employee performance, leaving out other vital variables such as employee job satisfaction, organizational performance, and customer satisfaction. Also, the research investigates the impact of corporate culture and employee performance on workers’ stress. Precisely, it fails to consider other significant stressors that affect employee performance. Thus, the present study’s scope is limited to understanding the relationship between organizational culture, employee competency, and worker stress.

Additionally, there is limited information on the impact of organizational culture and employee competency on workers’ stress. Much information that exists is about the relationship between corporate culture and employee performance [1,2,3,63,80]. Notably, a lack of reliable data requires the researcher to limit the analysis scope. Also, limited information can be a substantial obstacle in determining a meaningful relationship between variables. In this regard, the lack of sufficient data made it challenging for the researcher to find a significant relationship between organizational culture, employee competency, and stress. Also, few studies have attempted to link employee competency with corporate culture and workers’ stress. Thus, this implies that previous researchers have not widely explored the topic of the current research. As a result, the precious researchers failed to retrieve valuable and more in-depth information from secondary sources. Notably, citing previous studies is vital in completing a comprehensive literature review. Also, prior researches form the basis for understanding a given research problem. In this case, limited studies prevented the researcher from gaining an in-depth understanding of whether organizational culture and employee competency affect workers’ stress. Therefore, the limited available information prevented the researcher from exhaustively exploring the research topic.

Another study limitation is that it focused on secondary data to explore the effect of organizational culture and employee competency on workers’ stress [81]. Although secondary data provides detailed insight into the research topic, it does not cover the perceptions of the employees and the management. As a result, this implies the study outcomes do not reflect how employees feel concerning stressors. Exploring the topic by collecting primary data can provide valuable information to understand employees’ perspectives regarding the impact of organizational culture and employee competency on workers’ stress. Conducting empirical research is crucial in generating useful information about the topic. Specifically, the current researchers failed to utilize qualitative research methods such as interviews, focus group discussions, and questionnaires to examine stress determinants among workers. Based on this, the current researchers did not obtain sufficient data on the research topic, limiting an understanding of stressors in the workplace that affect employee performance. Besides, the current research has not limited the search to a specific organization or industry. The lack of a particular sector implies a broad research scope, making it difficult to understand the research topic. Generally, the research limitations may limit the generalizability of the study outcomes; hence, there is a need to address them by future researchers.

### 6.2. Future Suggestion

Given the study limitation, there is a need for further research to address the highlighted issues. These statistical findings of the present study corroborate the existing researches [1,2,3,63] pertaining to the relationship between two organizational cultures and two employee competencies and thereby, suggests for future researchers theoretically that two different types of competencies organizations desire to build and two different types of cultures organizations have established is associated with employee’s perceived stress. That indicates that the present study contributes to the literature in HR compensation practices by synthesizing the extant researches and extending this study based on current statistical results and thus, advocating the firm’s employee stress management ought not to exclude employee competencies and organizational cultures as a component of analysis. Based on this contribution of the current study, the detailed future suggestions.

First, future studies should explore additional employee competencies, including innovation, technical expertise, and result orientation. The current research explores two employee competencies, which are adaptability and customer orientation. Expanding the scope and exploring all the five employee competencies can give valuable insight into the impact of worker competency on stress. Also, it is imperative to understand how these competencies relate to the clan and market organizational cultures. Every employee competency relates differently with the clan or market culture; hence, their effect on workers’ stress varies. Therefore, future studies should provide additional information regarding adaptability and customer orientation and give insight into the other employee competencies. The current research hypothesizes that employees who possess adaptability competency and work in organizations with clan culture tend to be less stressed than their counterparts in companies with market culture. Besides, it hypothesizes that employees with customer orientation competency and working in firms with market culture are less stressed than their peers in organizations with clan culture. Given this, further research is needed to cover how employees possessing innovation, result orientation, and technical expertise competencies feel while working in organizations with clan and market cultures. Thus, there will be adequate data on the relationship between employee competency and organizational culture and stress. As a result, this will lead to broader coverage of literature, allowing for an in-depth understanding of how different employee competencies affect workers’ stress.

Second, future researchers should investigate other variables like employee job satisfaction, customers’ satisfaction, and organizational performance [80] to provide insight into their relationship with the organizational culture and impact on worker’s stress. Employee performance is just one of the factors affected by the organizational culture. Corporate performance, employee and customer satisfaction also relate to organizational culture and impact worker’s stress differently. Likewise, further research is needed to investigate other causes of stress on employees and how they affect worker performance. Precisely, employee performance is based on job satisfaction and organizational support, implying that lack of support and satisfaction causes stress and reduced performance. Thus, expanding the research scope to include these variables can help individuals to gain a comprehensive understanding of how various factors influence workers’ stress.

Additionally, the current research relied on prior studies [1,2,3,63,65] as an instrument to understand the research topic. In this regard, future studies should adopt additional tools or studies to determine the link between employee competence, organizational culture, and worker stress. Such research will provide similar outcomes to the current study’s findings, thus reinforcing the relationship between the three variables. Therefore, future researchers should extensively research the topic by exploring many prior studies to enrich secondary data.

Further, future studies should examine how different jobs are affected by employee competencies within the organization. Precisely, future researchers should aim at determining whether job positions control workers’ competencies. Hence, future studies need to examine if there is a correlation between organizational culture, employee competency, and job stress, seeking the generalizability of the outcomes. Precisely, future research may focus on empirically testing the relationships between these factors to understand if they can be generalized to the broad spectrum of organizations. Generally, future studies should focus on providing additional information critical to understanding the issue under investigation and reinforcing the current research outcomes.

## 7. Conclusions

The current research hypothesized that employees with advanced adaptability competence in a clan culture have lower levels of workplace stress than the employees within a market corporate culture. Based on the outcomes of previous studies [1,2,63], it is evident that clan culture supports employee adaptability competency. Precisely, employees that possess adaptability competency are more flexible and can adapt to any changes in the work environment. Besides, the clan culture promotes knowledge creation and proactive behavior among employees, enabling them to cope with the changes within an organization. Thus, employees working in a clan culture organization are less stressed than those working in the market organizational culture. Prior studies [1,2,3,63], also confirm that a market corporate culture does not support adaptability competency, making it difficult for employees to adapt to changes within the company. As a result, this increases stress and affects their job performance. Therefore, the current research confirms the hypothesis that employees with adaptability competence in a clan culture have lower levels of workplace stress than their counterparts in a market corporate culture. Moreover, the current study confirms that greater customer orientation competency among employees within a market culture makes the employees less stressed. Workers in organizations with market culture are less stressed than those with clan culture due to greater customer orientation competency. In this regard, market culture promotes employee customer orientation competency, allowing employees to handle workplace stressors accordingly. Thus, the current research holds that market organizational culture enhances employee customer orientation competency, which reduces work-related stress.

Organizational culture is essential in improving employee performance [63,82]. Precisely, it delineates task completion and allows for employee interaction within the company. Employee performance relies on the firms’ ability to execute clear priorities. A company’s culture enhances employee performance by stressing common values and goals. Organizations with solid cultures observe their values and engage employees, which motivates them to perform better. Many researchers have explored the relationship between organizational culture and employee performance [1,3,80,82]. The outcomes of these studies reveal a positive correlation between increased employee performance and corporate culture. Employees are critical players in organizational success. Likewise, organizations with strong cultures create a favorable work environment that allows employees to improve their performance. Positive corporate cultures involving compensation, rewards, management support, training, and growth opportunities facilitate improved employee performance. Besides, organizations with robust cultures promote employee performance by reducing turnover and absenteeism and increasing productivity. Therefore, prior studies have found a meaningful relationship between organizational culture and worker performance.

Employee adaptability competency positively relates to clan organizational culture. Organizations with clan cultures are flexible, action-oriented, and adaptable to change, enabling employees to feel less stressed and easily cope with any changes within the company. Contrarily, the market organizational culture negatively relates to adaptability competency. Organizations with a market culture prioritize control and stability. Also, they emphasize reaching targets and achieving results. Therefore, the pressures involved in market cultures make such organizations unsupportive of employee adaptability competency. Employee customer orientation competency positively relates to market culture. Precisely, employee market orientation relies on market culture effectiveness. Such organizational culture focuses on delivering excellent customer value to exceed client demands; thus, supporting customer orientation competency. However, it is negatively related to clan corporate culture since it emphasizes teamwork and flexibility and does not encourage employees to understand and meet consumers’ changing needs. Stress in the workplace is a common problem that affects many employees in terms of performance, productivity, and emotional well-being. In this regard, managing stress is necessary to employee performance and productivity.

## Figures and Tables

**Table 1:** Summarized Variable based on Previous Resources.

Main Constructs	Number of Questions	Prior Research
Clan Organizational Culture Market Organizational Culture	1–6 7–12	(Kang & Lee, 2021; Kerr & Slocum, 2005)
Adaptability Competency Customer Orientation Competency	13–18 19–22	(Díaz-Fernández et al., 2013; Kang & Lee, 2021)
Perceived Employee Stress	23–32	(Reis et al., 2010)

**Table 2:** Variable Information of the Current Research.

Main Factors	Description
Organizational Culture	The company is a personal place, it is like an extended family, People seem to share a lot of themselves. The leadership in the company is generally considered to exemplify mentoring, facilitating, or nurturing. The management style in the company is characterized by teamwork, consensus and participation. The ‘glue’ that holds the company together is loyalty and mutual trust. Commitment to the company runs high. The company emphasizes human development. High trust, openness and participation persist. The company defines success on the basis of the development of human resources, teamwork, employee commitment and concern for people. The Company is results orientated. A major concern is with getting the job done. People are very competitive and achievement orientated. The leadership in the company is generally considered to exemplify a no-nonsense, aggressive, results-orientated focus. The management style in the company is characterized by hard-driving competitiveness, high demands and achievement. The ‘glue’ that holds the company together is the emphasis on achievement and goal accomplishment. The company emphasizes competitive actions and achievement. Hitting stretch targets and winning in the marketplace is dominant. The company defines success on the basis of winning.
Employee Competency	13. Your task is supposed to handle smoothly multiple demands, shifting priorities, and rapid change 14. Your task is supposed to adapt responses and tactics to fit fluid circumstances 15. Your task is supposed to be flexible in how you see events 16. Your task should be very responsive and changes easily. 17. Your job is supposed to respond well to competitors and other changes in the business environment. 18. You need to continually adopt new and improved ways to do work. 19. Your task is supposed to understand customers’ needs and match them to services or products. 20. Your task is supposed to seek ways to increase customers’ satisfaction and loyalty. 21. Your task is supposed to gladly offer appropriate assistance. 22. You need to grasp a customer’s perspective, acting as a trusted advisor.
Perceived Employee Stress	25. Have you been upset because of something that happened unexpectedly? 26. Were you unable to control the important things in your life? 27. Did you feel nervous and ‘stressed’? 28. Are you confident about your ability to handle your personal problems? ^®^ 29. Do you feel that things were going your way? ^®^ 30. Did you find that you could not cope with all the things that you had to do? 31. Have you been able to control irritations in your life? ^®^ 32. Did you feel that you were on top of things? ^®^ 33. Have you been angered because of things that were outside of your control? 34. Do you feel difficulties were piling up so high that you could not overcome them?

**Table 3:** Procedure of Data Collection.

	Total	%
Survey Distributed	800	100
Uncollected Survey	133	16.6
Collected Survey	667	83.4
Discarded Survey	26	3.25
Usable Survey	641	80.1

**Table 4:** Demographic information.

	Number of Respondents	%
Job Level		
Managerial Position	303	47.3
Non-Managerial Position	338	52.7
Industry		
Manufacturing Sector	331	53.2
Service Sector	310	46.8
Age distribution		
20s	77	12.0
30s	80	12.5
40s	105	16.4
50s	59	9.2
Over 50s	69	11.4
Final Education Level		
High School	91	14.2
Associate	136	21.2
Bachelor	213	33.2
Master	155	24.2
Doctoral	46	7.2
Gender		
Male	298	46.5
Female	343	53.5

**Table 5:** Descriptive Statistics for Final Sample.

Factors	Mean	Median	Range (Max–Min)	Std. D
Clan Culture	4.11	4.43	6 (7–1)	0.754
Market Culture	3.72	3.92	6 (7–1)	0.839
Adaptability Competency	3.29	3.55	6 (7–1)	0.442
Customer-Orientation Competency	3.34	3.62	6 (7–1)	0.457
Perceived Employee Stress	3.80	3.98	6 (7–1)	0.782

**Table 6:** Descriptive Statistics for Final Sample.

Factors	Mean Value of Clan Culture	Mean Value of Market Culture
Group 1: Samples (*n* = 308) above the mean value (3.29) of Adaptability	5.22	3.23
Group 2: Samples (*n* = 333) below the mean value (3.29) of Adaptability	3.47	4.42
Group 3: Samples (*n* = 352) above the mean value (3.34) of Customer-orientation	2.66	4.98
Group 4: Samples (*n* = 289) below the mean value (3.34) of Customer-orientation	3.95	2.97

**Table 7:** The Statistical findings of reliability investigation.

Sub-Factors	Question	The Value of Cronbach’s α
Clan Organizational Culture	1–6	0.676
Market Organizational Culture	7–12	0.707
Adaptability Employee Competency	13–18	0.757
Customer-Orientation Competency	19–22	0.669
Perceived Employee Stress	23–32	0.803

**Table 8:** The Statistical Results of CFA.

Variables	Unstandardized Factor Loadings	Standardized Factor Loadings	S.E.	C.R.	AVE	Construct Reliability
CIAN1	1.00	0.90				
CIAN2	0.71	0.68	0.5	15.12 ***		
CIAN3	0.83	0.81	0.5	16.43 ***	0.837	0.798
CIAN4	0.77	0.73	0.5	16.31 ***		
CIAN5	0.90	0.81	0.5	18.13 ***		
CLAN6	0.72	0.69	0.5	16.51 ***		
MARKET1	1.00	0.81				
MARKET2	0.79	0.67	0.5	15.65 ***		
MARKET3	0.81	0.62	0.5	17.54 ***	0.797	0.772
MARKET4	0.84	0.76	0.5	18.24 ***		
MARKET5	1.03	0.89	0.5	16.61 ***		
MARKET6	0.80	0.83	0.5	16.58 ***		
ADAPT1	1.00	0.90				
ADAPT2	0.88	0.80	0.5	19.63 ***		
ADAPT3	1.02	0.89	0.5	20.11 ***	0.754	0.711
ADAPT4	0.97	0.82	0.5	22.53 ***		
ADAPT5	0.76	0.72	0.5	18.39 ***		
ADAPT6	0.86	0.78	0.5	19.17 ***		
COC1	1.00	0.92				
COC2	0.86	0.81	0.4	16.85 ***		
COC3	0.71	0.67	0.4	19.34 ***	0.783	0.739
COC4	0.88	0.79	0.4	20.13 ***		
Stress1	1.00	0.88				
Stress2	0.71	0.67	0.5	15.67 ***		
Stress3	0.73	0.70	0.5	16.86 ***		
Stress4	0.70	0.66	0.5	19.15 ***		
Stress5	0.68	0.62	0.5	17.78 ***		
Stress6	0.74	0.71	0.5	17.49 ***	0.741	0.711
Stress7	0.80	0.74	0.5	18.11 ***		
Stress8	0.77	0.72	0.5	19.47 ***		
Stress9	0.69	0.67	0.5	16.13 ***		
Stress10	0.81	0.77	0.5	18.17 ***		

*** *p* < 0.00.

**Table 9:** The Results of Discriminant Validity.

Construct	1	2	3	4	5
1. Clan Culture	0.765				
2. Market Culture	0.348	0.802			
3. Adaptability	0.719	0.479	0.725		
4. Customer-orientation	0.436	0.761	0.464	0.843	
5. Perceived Stress	0.562	0.521	0.484	0.497	0.719

**Table 10:** The main result of the research model.

Route	Unstandardized Coefficients	Standardized Coefficients (β)	S.E.	T
Group 1 and Perceived Stress	0.36	0.31	0.06	0.529 ***
Group 2 and Perceived Stress	0.51	0.48	0.05	0.635 ***
Group 3 and Perceived Stress	0.38	0.33	0.07	0.532 ***
Group 4 and Perceived Stress	0.46	0.51	0.05	0.562 ***

<χ^2^ = 243.32 (*df* = 95, *p* < 0.001)), TLI = 0.951, RMR = 0.017, CFI = 0.955, GFI = 0.913, RMSEA = 0.061 (90% CI: From 0.044 to 0.063) *** *p* < 0.001.

## Data Availability

Used data for this study is not available.
